# Androgen Receptor-Mediated Nuclear Transport of NRDP1 in Prostate Cancer Cells Is Associated with Worse Patient Outcomes

**DOI:** 10.3390/cancers13174425

**Published:** 2021-09-02

**Authors:** Thomas Steele, Anhao Sam, Shawna Evans, Elizabeth Browning, Sheryl Krig, Katelyn Macias, Adarsh Konda, Salma Siddiqui, Blythe Durbin-Johnson, Paramita Ghosh, Ruth Vinall

**Affiliations:** 1 Department of Pharmaceutical & Biomedical Sciences, California Northstate University College of Pharmacy, Elk Grove, CA 95757, USA; thomas.steele@cnsu.edu (T.S.); anhao.sam@cnsu.edu (A.S.); shawnajevans@gmail.com (S.E.); elizabeth1browning@gmail.com (E.B.); srkrig@gmail.com (S.K.); katelyn.macias@hotmail.com (K.M.); AdarshKondaRx@gmail.com (A.K.); 2 Department of Urological Surgery, School of Medicine, University of California Davis, 4860 Y Street, Sacramento, CA 95817, USA; paghosh@ucdavis.edu; 3 Research Service, VA Northern California Healthcare System, 10535 Hospital Way, Mather, CA 95655, USA; SALMA.SIDDIQUI@va.gov; 4 Department of Public Health Sciences, School of Medicine, University of California Davis, One Shields Avenue, Davis, CA 95616, USA; bpdurbin@ucdavis.edu; 5 Department of Biochemistry and Molecular Medicine, School of Medicine, University of California Davis, 2700 Stockton Blvd, Sacramento, CA 95817, USA

**Keywords:** NRDP1, prostate cancer (CaP), androgen receptor

## Abstract

**Simple Summary:**

NRDP1 is an E3 ubiquitin ligase that has been shown by our group and others to target ErbB3 for proteasomal degradation in prostate and breast cancer cells and thereby decrease the likelihood cancer progression. Our group has found that NRDP1 can be located in the nucleus as well as the cytoplasm of prostate cancer (CaP) cells, which is unexpected as NRDP1 lacks a nuclear localization signal. Here we elucidate the mechanism by which nuclear translocation of NRDP1 can occur and demonstrate that nuclear NRDP1 retains its ubiquitin ligase activity. Our patient data and cell line studies indicate that increased levels of nuclear NRDP1 contributes CaP progression, thereby underscoring the clinical relevance of our findings and supporting continued investigation and elucidation of the specific role(s) played by NRDP1 in the nucleus of CaP cells.

**Abstract:**

To our knowledge, our group is the first to demonstrate that NRDP1 is located in the nucleus as well as the cytoplasm of CaP cells. Subcellular fractionation, immunohistochemistry, and immunofluorescence analysis combined with confocal microscopy were used to validate this finding. Subcellular fractionation followed by western blot analysis revealed a strong association between AR and NRDP1 localization when AR expression and/or cellular localization was manipulated via treatment with R1881, AR-specific siRNA, or enzalutamide. Transfection of LNCaP with various NRDP1 and AR constructs followed by immunoprecipitation confirmed binding of NRDP1 to AR is possible and determined that binding requires the hinge region of AR. Co-transfection with NRDP1 constructs and HA-ubiquitin followed by subcellular fractionation confirmed that nuclear NRDP1 retains its ubiquitin ligase activity. We also show that increased nuclear NRDP1 is associated with PSA recurrence in CaP patients (n = 162, odds ratio; 1.238, *p* = 0.007) and that higher levels of nuclear NRDP1 are found in castration resistant cell lines (CWR22Rv1 and PC3) compared to androgen sensitive cell lines (LNCaP and MDA-PCa-3B). The combined data indicate that NRDP1 plays a role in mediating CaP progression and supports further investigation of both the mechanism by which nuclear transport occurs and the identification of specific nuclear targets.

## 1. Introduction

Ubiquitin ligases play a key role in the post-translational regulation of cellular protein levels and their dysregulation has been shown to contribute to the development and/or progression of several cancer types including gliomas and breast and prostate cancers [1,2]. Ubiquitin ligases make attractive drug targets since they can regulate expression of several tumor suppressors and oncogenes which drive carcinogenesis. For example, expression levels of PTEN, AKT, mTOR, c-Myc and p53 can all be regulated via ubiquitination [3,4]. NRDP1 is an E3 ubiquitin ligase which has been implicated in the progression of gliomas and prostate, breast, and liver cancers [5,6,7,8]. Our group and others have shown that ErbB3, a tyrosine kinase receptor which can drive prostate and breast cell proliferation, is key target of NRDP1; decreased NRDP1 expression causes elevation of ErbB3 levels and can thereby increase prostate and breast cancer cell proliferation and survival [7,9,10]. It is noteworthy that NRDP1 also plays a key role in the development and progression of several neurological diseases including Alzheimer’s and Parkinson’s disease [11,12] by promoting neuronal cell apoptosis [13,14]. An increased understanding of NRDP1 biology has the potential to improve our understanding of disease pathogenesis and support the development of new treatments for these neurological diseases as well as for prostate cancers and other cancer types.

Our group has previously demonstrated that NRDP1 can be transcriptionally regulated by AR in CaP cells; activated AR is able to bind to the NRDP1 promoter region and drive NRDP1 expression [7,15]. This was observed in androgen sensitive but not castration resistant CaP (CRPC) cell lines indicating that dysregulation of NRDP1 expression may contribute to disease progression. We also found a strong association between NRDP1 and AR expression levels in biopsies from patients with localized disease. Unexpectedly, NRDP1 staining was detected in the nucleus of CaP biopsy cells as well as in the cytoplasm; nuclear localization was unexpected since NRDP1 does not have a nuclear localization signal. To our knowledge, expression of NRDP1 in the nucleus of CaP cells has not previously been reported and the mechanism(s) by which nuclear translocation occurs, the function of nuclear NRDP1, and its clinical relevance remain completely unknown.

The main goals of the current study were to validate our finding that NRDP1 can be located in the nucleus of CaP cells, to elucidate the mechanism by which nuclear translocation occurs, and to determine whether nuclear NRDP1 retains its ubiquitin ligase activity. We also conducted experiments to determine how nuclear NRDP1 levels are regulated and conducted patient studies to establish whether levels of nuclear NRDP1 in biopsy samples from CaP patients with localized disease are associated with disease progression.

## 2. Materials and Methods

### 2.1. Patient Characteristics

All data were collected with approval from the VA Northern California Health Care System (VANCHCS) Institutional Review Board. Sections from formalin fixed paraffin-embedded prostate tumors of 162 patients who underwent prostatectomy at VANCHCS were analyzed for these studies. Patient characteristics are described in Table 1. Tumor areas were identified by a pathologist and 60 µm core samples were extracted. Specimens were arranged in triplicate in a tissue microarray (TMA) using a Beecher Instruments Manual Tissue Arrayer (Sun Prairie, WI, USA). Hematoxylin-eosin staining was used as a reference for interpreting the additional sections of the TMA. Immunohistochemical analyses using Ki67, NRDP1, and AR antibodies was performed as previously described [16].

### 2.2. Reagents

Primary antibodies; Androgen receptor (AR, cat#3202S), tubulin (cat#9F3), Lamin A/C (cat#2032), and HA-TAG (cat#3724S) primary antibodies (Cell Signaling Technology, Danvers, MA, USA), NRDP1 (Santa Cruz Biotechnology, Dallas, TX, USA, cat#sc-365622), GAPDH (Sigma-Aldrich, St. Louis, MO, USA, cat#39-8600), FLAG (Genscript, Piscataway, NJ, USA, cat#A00187-200). Secondary antibodies; HRP-linked anti-mouse IgG (Cell Signaling Technology, Danvers, MA, USA, cat#7076), HRP-linked goat anti-rabbit secondary (Jackson Immunoresearch, West Grove, PA, USA, cat#111-035-144). siRNA; AR siRNA was a pool of 4 duplexes with the following sequences; #1: 5′-CAGUCCCACUUGUGUCAAATT-3′, #2: 5′-CUGAUCUGUGGAGAUGAATT-3′, #3: 5′-GUCGUCUUCGGAAAUGUUATT-3′, #4: 5′-GACAGUGUCACACAUUGAATT-3′ (Santa Cruz Biotechnology, Dallas, TX, USA). Control siRNA was a pool of 4 scrambled non-specific siRNA duplexes (Santa Cruz Biotechnology, Dallas, TX, USA). Drugs; R1881 (Sigma-Aldrich, St. Louis, MO, USA), Enzalutamide, Cycloheximide, MG132 (Selleckchem, Houston, TX, USA).

### 2.3. Cell Lines and Culture

The following CaP cell lines were used; LNCaP, 22Rv1, MDA-PCa-2B and PC3 (ATCC, Manassas, VA, USA). LNCaP, 22Rv1, and PC3 were cultured in RPMI1640 media (Invitrogen/GIBCO, Carlsbad, CA, USA) supplemented with 10% fetal bovine serum (FBS, Omega Scientific, Inc., Tarzana, CA, USA), 2 mM L-glutamine, and 100 U/mL penicillin-100 μg/mL streptomycin (Invitrogen/GIBCO, Carlsbad, CA, USA). MDA-PCa-2B were cultured in F-12K media supplemented with 20% FBS (Omega Scientific, Inc., Tarzana, CA, USA), 2 mM L-glutamine, and 100 U/mL penicillin-100 μg/mL streptomycin (Invitrogen/GIBCO, Carlsbad, CA, USA), 25 ng/mL cholera toxin, 100 pg/mL hydrocortisone (Sigma-Aldrich, St. Louis, MO, USA), 10 ng/mL mouse Epidermal Growth Factor, 0.005 mM phosphoethanolamine, 45 nM sodium selenite, and 0.005 mg/mL human recombinant insulin (Thermofisher, Waltham, MA, USA). Cells were kept at 37 °C in a humidified environment of 5% CO_2_ in air.

### 2.4. Immunofluorescence and Confocal Microscopy

Cells were fixed in ice cold methanol for 10 min then washed with PBST and blocked using 10% BSA in PBST prior to being incubated with primary antibodies overnight at 4 °C. Cells were then washed and incubated with either Alexa Fluor 488 conjugated goat anti-rabbit IgG secondary antibody or Alexa Fluor 555 conjugated goat anti-rabbit IgG secondary antibody (Thermofisher, Waltham, MA, USA) for 1.5 h before being washed again and mounted using Ultra Cruz hard-set mounting medium with DAPI (Santa Cruz Biotechnology, Dallas, TX, USA). Representative fluorescence images were taken on a Leica DMR. Confocal images were taken on an Olympus FluoView FV1000 confocal microscope (Olympus Scientific Solutions, Waltham, MA, USA).

### 2.5. Subcellular Fractionation

Subcellular fractionation was performed as previously described [17]. Briefly, cell culture dishes were washed with two to three times with PBS and lysed in cytoplasmic lysis buffer before being spun at 16,000× *g* for 5 min to isolate the cytoplasmic fraction. The remaining nuclear pellet was washed three times in cytoplasmic lysis buffer before being lysed in 1× SDS sample buffer.

### 2.6. Transfections and Plasmids

Lipofectamine 2000 (Invitrogen, Carlsbad, CA, USA) was used for all transfections as per manufacturer’s instructions. Cells were plated in 100 mm or 150 mm dishes and allowed to attach overnight prior to transfection with siRNA or plasmid constructs. Plasmid constructs; pcDNA3, Wt-NRDP1-FLAG, CSHQ-NRDP1-FLAG, ΔCC-NRDP1-FLAG, Ubiquitin-HA-TAG, Wt-AR, AR-V7 (missing the ligand binding domain), AR-ΔDBD (missing residues 538–614 of the DNA binding domain (‘DBD’)), AR-NTD, pEL 77 AR (missing residues 647–670 of the hinge domain (‘Hinge 1’)), and AR w/o (missing residues 628–646 of the hinge domain). Cells were transfected with a total of 3.0 µg of plasmid per 100 mm or 150 mm dish. Wt-NRDP1-FLAG, CSHQ-NRDP1-FLAG (harbors two-point mutations (C34S, H36Q) in the receptor binding region (RING finger domain), ΔCC-NRDP1-FLAG (missing the coiled coil domain), and Ubiquitin-HA-TAG were kindly provided by Dr. Kermit Carraway III. AR-V7 was kindly provided by Dr. Allen Gao. Wt-AR, AR-ΔDBD, and AR-ΔNTD were from Addgene (Addgene, Watertown, MA, USA).

### 2.7. Protein Extraction and Western Blot

Protein extraction and western blot analyses were performed as previously described [18]. Briefly, whole cell lysates were collected on ice and washed three times with PBS and lysed with SDS sample buffer for 15 min. Protein content was quantified with a BCA Protein assay kit from Pierce (Pierce Biotechnology, Waltham, MA, USA). SDS-PAGE gels were run and transferred with Bio-Rad apparatus (Bio-Rad, Hercules, CA, USA) onto 0.2 micron-pore Millipore Sigma PVDF membranes (Sigma-Aldrich, St. Louis, MO, USA). Membranes were blocked with 5% milk, incubated with primary antibody overnight at 4 °C, incubated with secondary for 1.5 h, and washed in TBST between each step. Membranes were developed by incubating in Pierce Supersignal West Femto Maximum Sensitivity Substrate (Pierce Biotechnology, Waltham, MA, USA) and developed with x-ray film or a GE Amersham 680 Imager (GE Healthcare, Chicago, IL, USA).

### 2.8. Immunoprecipitation

Cells were plated in 100 mm or 150 mm dishes and lysed in ice cold IP lysis buffer (Pierce Biotechnology, Waltham, MA, USA). Protein was quantified with BCA protein assay (Pierce Biotechnology, Waltham, MA, USA). One milligram of protein was incubated with 4 ug of each primary antibody overnight at 4 °C followed by incubation with protein A agarose beads (Cell Signaling Technology, Danvers, MA, USA) overnight at 4 °C. The complex was pelleted and washed three to five times with IP lysis buffer and then eluted with SDS-sample buffer with BME and run on SDS-PAGE gels.

### 2.9. Statistical Analyses

Cell line data; at least three independent experiments were completed for each analysis described in this article. Patient data; correlations between markers were calculated using the Spearman correlation. Recurrence-free percentages at five years for low and high marker expression were calculated from Kaplan-Meier curves. The association between marker expression and PSA failure was analyzed using logistic regression models. Analyses of patient data were conducted using R version 3.6.0 (accessed on 24 April 2019).

## 3. Results

### 3.1. NRDP1 Protein Is Expressed in the Nucleus as Well as the Cytosol in Prostate Cancer Cell Lines

**Figure 1:**
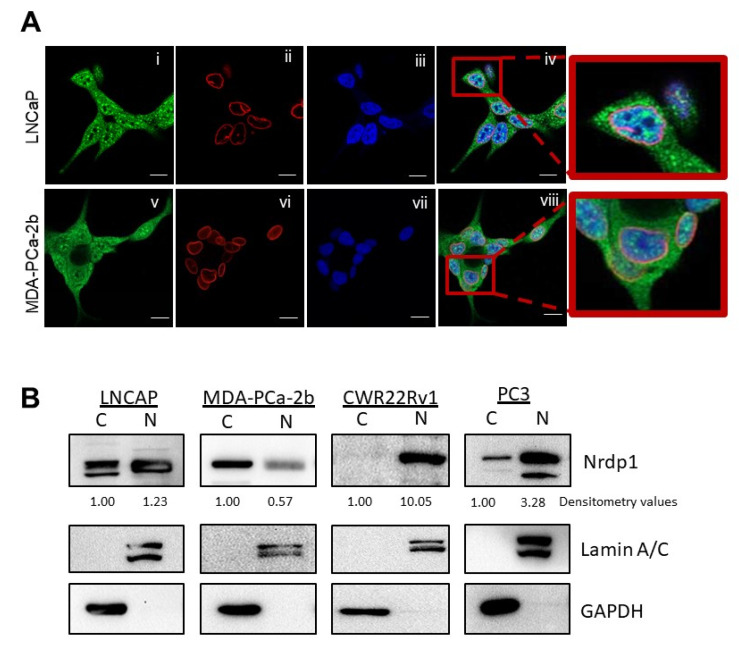
NRDP1 protein is expressed in the nucleus as well as the cytoplasm in prostate cancer cell lines. Confocal microscopy analysis of NRDP1 immunofluorescence staining in LNCaP and MDA-Pca-2b cell lines (**A**) revealed that NRDP1 is located in both the nucleus and cytoplasm of prostate cancer cells. Localization of NRDP1 in the nucleus of prostate cancer cells has not previously been reported. NRDP1 appeared as distinct puncta in the cytoplasm and nucleus. In LNCaP cell (**i**–**iv**), more puncta were observed and were brighter compared to those observed in MDA-Pca-2b cells (**v**–**viii**) indicating higher levels of expression in LNCaP. Subcellular fractionation followed by western blot (**B**) confirmed that NRDP1 is located in the nucleus of LNCaP and MDA-Pca-2b cells as well as CWR22Rv1 and PC3 cells. Higher levels of nuclear NRDP1 relative to cytoplasmic NRDP1 were observed in CWR22Rv1 and PC3 cell lines (both castration resistant cell lines, cytoplasmic:nuclear NRDP1 ratios were 1:10.05 and 1:3.28, respectively) compared to LNCaP and MDA-Pca-2b cell lines (both androgen sensitive cell lines, 1:1.23 and 1:0.57, respectively). Detailed information about Western Blot can be found at supplementary materials. Green = NRDP1, Red = nuclear lamin, Blue = DAPI (nucleus) scale bar = 10 µm.

We previously demonstrated androgen receptor (AR) regulation of NRDP1 in hormone-naïve CaP [7], and that NRDP1 levels correlated with AR in localized human prostatectomy specimens [15]. NRDP1 was observed in the nucleus of some CaP cells in the patient prostatectomy specimens. This was completely unexpected as NRDP1 does not have a nuclear localization signal and the presence of NRDP1 in the nucleus of CaP cells had not previously been reported. To confirm this observation, we conducted immunofluorescent analysis of two cell lines–LNCaP (mutant AR T877A) and MDA-PCa-2b (mutant AR T877A, L701H) [19], using an anti-NRDP1 antibody (Figure 1A). NRDP1 was observed in both the cytoplasm and in the nucleus of these cells. Confocal microscopy revealed that NRDP1 localizes in puncta distributed throughout the cytoplasm and nucleoplasm in both LNCaP and MDA-PCa-2b cells (Figure 1A). Nuclear NRDP1 did not appear to be located within nucleoli.

To determine whether NRDP1 is also expressed in the nuclei of castration resistant CaP (CRPC), we broadened our investigation to two CRPC cell lines-PC3 (AR null), and CWR22Rv1 (mutant AR H874Y; exon 3 duplication; splice variants). The presence of NRDP1 in the nucleus of all four CaP cells was validated by subcellular fractionation studies (Figure 1B, full blot; Figure S6), which revealed that CRPC cell lines (PC3 and CWR22Rv1) express higher levels of nuclear NRDP1 compared to androgen sensitive cell lines (LNCaP and MDA-PCa-2b); cytoplasmic to nuclear ratio of NRDP1 was 1:0.57 in MDA-PCa-2b cells and 1:1.23 in LNCaP cells compared to 1:10.05 in CWR22Rv1 cells and 1:3.28 in PC3 cells (Figure 1B). Taken together, these data demonstrate that NRDP1 is expressed in both the nucleus and the cytoplasm of CaP cell lines and that CRPC cell lines express relatively higher levels of nuclear NRDP1 compared to androgen sensitive cell lines. Again, nuclear localization of NRDP1 in CaP has not previously been reported.

### 3.2. Increased Levels of Nuclear NRDP1 in Patient Tumors from Prostatectomy Samples Are Associated with Worse Patient Outcome

**Figure 2:**
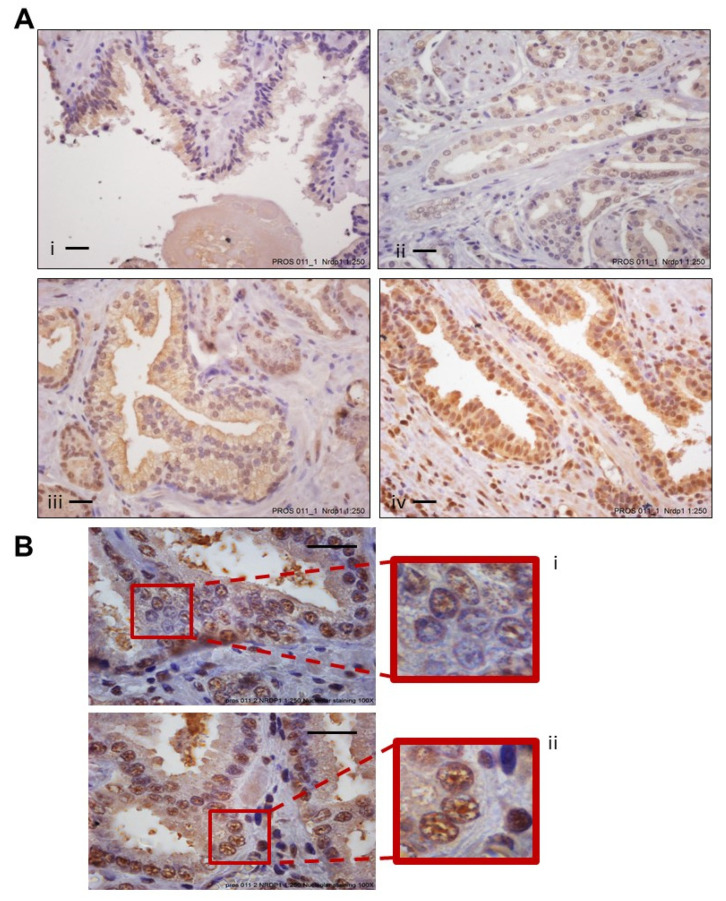
NRDP1 protein is also expressed in the nucleus of prostate cancer patient tumor cells. Immunohistochemical (IHC) analysis determined that NRDP1 is also located in the nucleus in prostate cancer patient cells (**A**,**B**). Patient samples varied in the degree of cytoplasmic:nuclear NRDP1 staining that was observed. For example, in some patients there was low cytoplasmic/low nuclear levels (**Ai**), intermediate cytoplasmic/intermediate nuclear levels (**Aii**), high cytoplasmic/low nuclear levels (**Aiii**), or high cytoplasmic/high nuclear levels (**Aiv**) of NRDP1. Higher magnification images (**Bi**,**Bii**) demonstrated that nuclear NRDP1 appeared as distinct puncta in the nucleus and this aligns with what was observed in prostate cancer cell lines. Scale bar = 50 µm. Brown = NRDP1, Blue = Hematoxylin (nucleus).

Nuclear localization of NRDP1 was also observed in patient prostatectomy sample tumor cells confirming our in vitro findings and indicating potential clinical relevance (Figure 2A,B). Table 1 shows patient characteristics. Significantly, high magnification pictures (Figure 2B) demonstrated similar punctate localization of NRDP1 in the nucleus as seen in the confocal microscopy pictures (Figure 1A). Additionally, we analyzed the expression of AR and the proliferation index Ki67 in the same tissues. Logistic regression analysis determined that in this patient cohort, higher levels of nuclear NRDP1 are associated with significantly higher odds of PSA failure; odds ratio of 1.238 (95% CI: 1.063, 1.443), *p* = 0.007 (Table 2). Kaplan-Meier analysis further determined that the recurrence-free percentage at five years following diagnosis for patients whose nuclear NRDP1 levels were below the median level versus above the median level was 73.5% versus 92%, a difference of −18.5% (*p* = 0.003), demonstrating that higher nuclear NRDP1 levels predict worse outcome (Table 3). An association between Nrdp1 (nuclear or cytoplasmic) was not observed, however, as would be expected increased levels of nuclear AR and increased Ki67 expression did correlate with increased Gleason score (Figure S1). Our finding that increased nuclear NRDP1 levels is associated with worse disease outcomes aligns with our in vitro observation that castration resistant cell lines express higher levels of nuclear NRDP1 compared to androgen sensitive cell lines (Figure 1B).

### 3.3. Strong Association between Levels of Androgen Receptor and NRDP1 in Prostate Cancer Patient Tumor Samples

Dysregulation of AR, including by mutation and expression of splice variants, is well known to drive CaP progression [20,21]. The AR is active in the nucleus, and therefore, AR localization in the cytoplasm is considered to demonstrate inactive AR. We investigated a correlation between AR and the proliferation index Ki67 with NRDP1 localization. As expected, based on data from multiple publications, there was a strong association between nuclear AR levels and Ki67 in all subgroups (Table 4 and Table 5) [22,23,24,25,26]. A stronger association was observed in patients with stage 1/2 CaP versus patients with stage 3 CaP; Spearman’s correlation coefficients were 0.22 (*p* = 0.012) and 0.51 (<0.001), respectively. No associations were found between nuclear NRDP1 and Ki67 indicating that nuclear NRDP1 does not directly contribute to increased proliferation of CaP patient tumor cells.

Correlation analysis revealed a strong negative association between levels of cytoplasmic AR and levels of nuclear NRDP1 in CaP patient samples (Table 4 and Table 5). In patients who were diagnosed with stage 1/2 (Table 4) or stage 3 (Table 5) CaP at time of prostatectomy, the Spearman’s correlation coefficient was −0.46 (*p* < 0.001) and −0.35 (*p* = 0.003), respectively. Association between nuclear AR and nuclear NRDP1 was not statistically significant, possibly due to NRDP-1-mediated degradation of nuclear Nrdp1. In support of this, ubiquitination of AR by other E3 ubiquitin ligases has previously been reported [27,28]. A negative association between cytosolic AR and nuclear NRDP1 was observed and this supports our in vitro data which indicate that AR and NRDP1 co-translate into the nucleus of CaP cells; unliganded cytosolic AR is unable to translocate to the nucleus since HSP90 blocks its nuclear localization signal [29,30] meaning this negative association would be expected.

### 3.4. Transport of NRDP1 into the Nucleus Is Associated with Nuclear Transport of the Androgen Receptor

**Figure 3:**
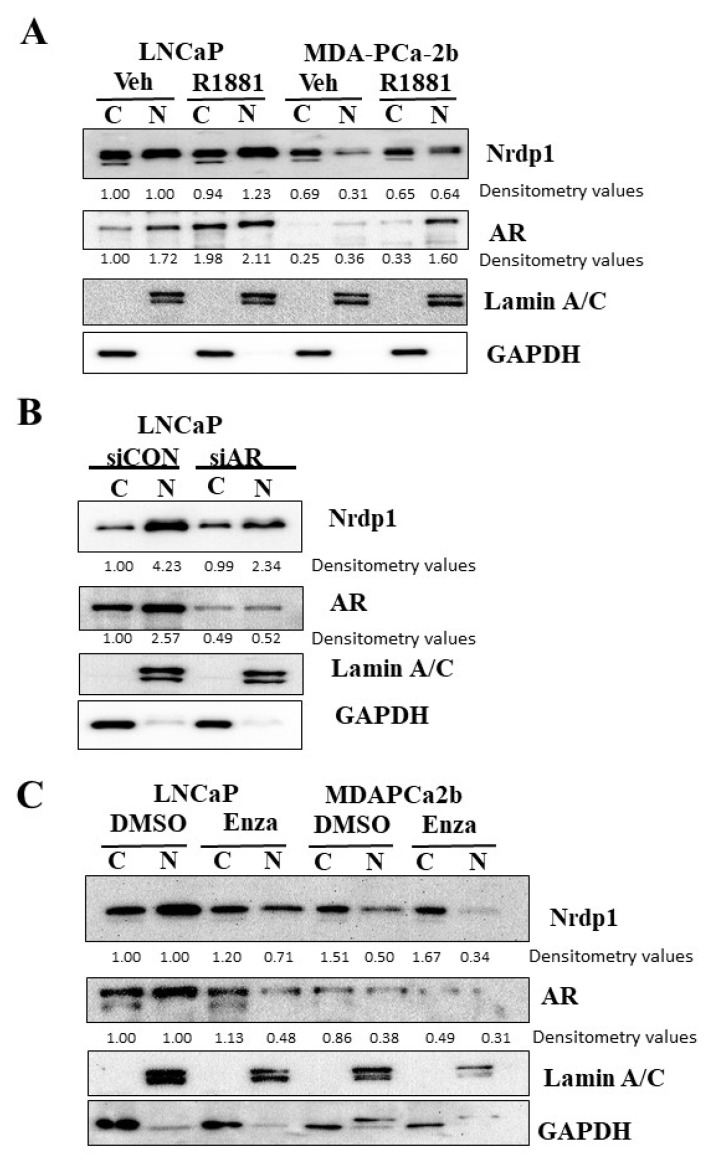
Transport of NRDP1 into the nucleus is associated with nuclear transport of the androgen receptor. Treatment of LNCaP and MDA-Pca-2b cells with synthetic androgen, R1881 (1 nM for 48 h) caused an increase in levels of both nuclear androgen receptor (AR) and nuclear NRDP1 (**A**) Inhibition of AR expression levels (treatment with AR siRNA, 50 nM for 48 h) (**B**) or inhibition of AR activity and translocation (treatment with Enzalutamide, 10 μM for 24 h) (**C**) caused a decrease in nuclear AR levels and a concurrent decrease in nuclear NRDP1 levels (**B**,**C**) The combined data demonstrate there is a strong correlation between AR and NRDP1 nuclear expression and translocation in these two cell lines. Detailed information about Western Blot can be found at supplementary materials.

Since NRDP1 does not contain a nuclear translocation signal, its transport into the nucleus must be dependent on another molecule(s). As AR activity correlated with NRDP1 nuclear localization in the primary tumors, we focused on determining whether the AR could play a role in nuclear transport of NRDP1. Binding of androgen to the AR promotes the transport of AR into the nucleus [31] and the AR has been shown to facilitate the nuclear transport of other molecules, for example beta-catenin [20,21]. Our data demonstrate that manipulation of AR nuclear expression and/or activation and translocation can impact nuclear NRDP1 levels in androgen sensitive LNCaP and MDA-PCa-2b cells. Treatment with the synthetic androgen R1881, a strong AR ligand, caused a 1.72-fold increase in nuclear AR levels and a 1.23-fold increase in nuclear NRDP1 levels in LNCaP cells, while it induced a 4.44-fold increase in nuclear AR levels and a concurrent 2.06-fold increase in nuclear NRDP1 in MDA-PCa-2b cells (Figure 3A, full blot; Figure S7). Converse results were observed when AR expression levels were decreased; inhibition of AR expression levels using an AR-specific siRNA caused a 4.94-fold decrease in nuclear AR levels and a concurrent 1.81-fold decrease in nuclear NRDP1 levels in LNCaP cells (Figure 3B, full blot; Figure S8). Inhibition of AR translocation with enzalutamide also caused reduced levels of nuclear NRDP1; treatment with enzalutamide resulted in a 2.08-fold decrease in nuclear levels of AR and a concurrent 1.41-fold decrease in nuclear levels of NRDP1 in LNCaP cells; while it caused a 1.23-fold decrease in nuclear AR and a 1.47-fold decrease in nuclear NRDP1 in MDA-PCa-2b (Figure 3C, full blot; Figure S9). Note that we confirmed our previous finding that AR can transcriptionally regulate Nrdp1 through reporter gene assay (Figure S2). The combined data clearly demonstrate that there is a strong correlation between AR activity and NRDP1 nuclear translocation in hormone sensitive CaP cell lines.

### 3.5. The Interaction between NRDP1 and AR Is Dependent on Presence of the AR Hinge Region but Does Not Require the NRDP1 Coiled Coil Domain

**Figure 4:**
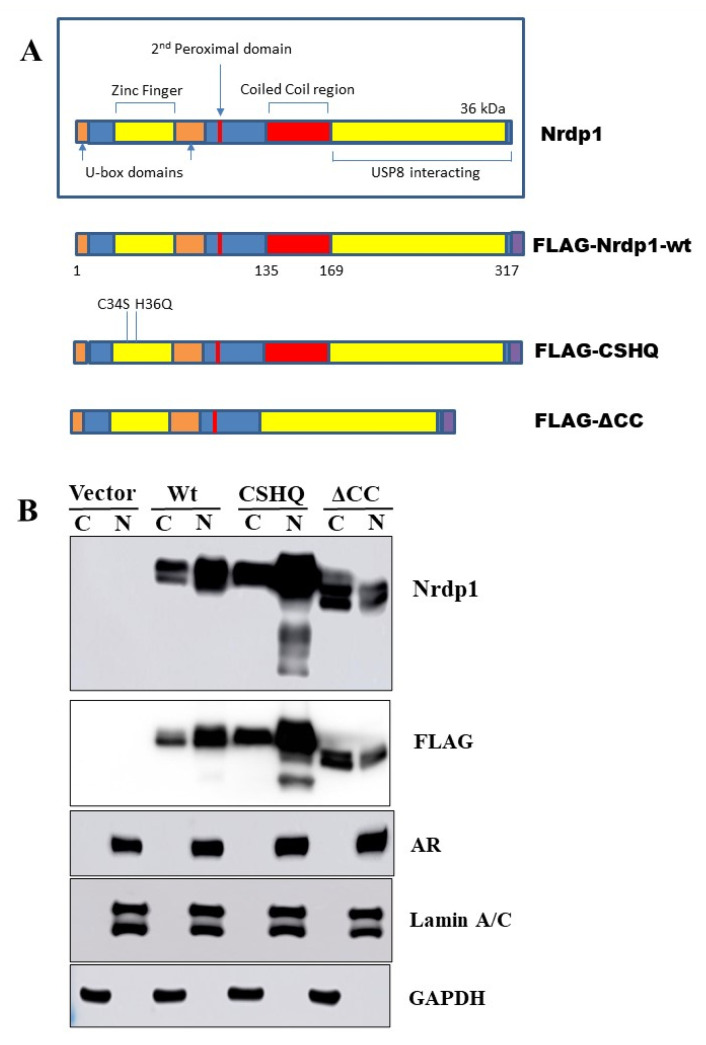
Overexpression of NRDP1 through transfection with various NRDP1 constructs caused high levels of nuclear NRDP1 expression. Three FLAG-tagged constructs were used to allow for manipulation of NRDP1 levels and to help determine whether interaction between AR and NRDP1 is possible; wildtype NRDP1 (Wt), NRDP1-CHSQ (CSHQ), and NRDP1-ΔCC (ΔCC) (**A**). The CSHQ construct harbors two point mutations in the receptor binding region (RING finger domain) which cause a dramatic reduction in the ability of NRDP1 to auto-ubiquitinate and to facilitate ubiquitination of target proteins. The ΔCC construct is missing the coiled coil domain this prevents NRDP1 oligomerization, a step that is required for efficient NRDP1 auto-ubiquitination to occur, however, ubiquitination of other NRDP1 target proteins by ΔCC is still possible. Transfection of LNCaP cells with these constructs (3 μg per 100 dish, 48 h) caused a dramatic increase in NRDP1 expression and high levels of nuclear NRDP1 were observed (**B**). Detailed information about Western Blot can be found at supplementary materials.

NRDP1 is an E3 ubiquitin ligase which can auto-ubiquitinate as well as ubiquitinate several target molecules including ErbB3 [32,33]. The RING finger domain (RING) facilitates the transfer of ubiquitin from E2 ubiquitin-conjugating enzymes onto NRDP1 target proteins, the coiled-coil domain allows for oligomerization and is required for NRDP1 autoubiquitination, and the receptor binding domain recruits NRDP1 targets [34]. Three NRDP1 plasmid constructs, all of which are FLAG-tagged, were used to help further elucidate the mechanism by which NRDP1 can translocate to the nucleus [32]. These are shown in Figure 4A; wildtype (WT) NRDP1, CHSQ (harbors two point mutations (C34S, H36Q) in the receptor binding region (RING finger domain) which cause a dramatic reduction in the ability of NRDP1 to auto-ubiquitinate and to facilitate ubiquitination of binding partners) [32], and ΔCC (the coiled coil domain is missing and this prevents oligomerization, a step that is required for efficient NRDP1 auto-ubiquitination to occur [34]. Note that ubiquitination of other NRDP1 binding partners by ΔCC is still possible).

**Figure 5:**
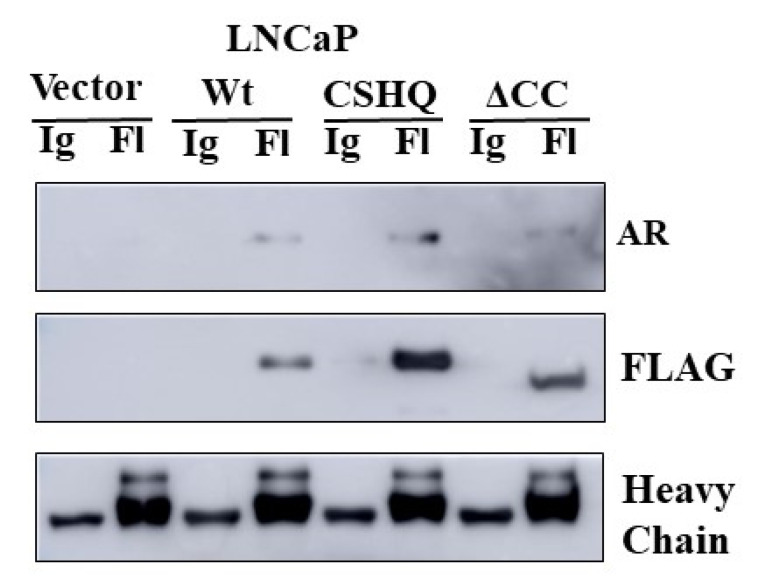
The interaction between NRDP1 and the androgen receptor does not require the NRDP1 coiled coil domain. Transfection with the Wt, CSHQ, or ΔCC FLAG-tagged NRDP1 constructs (3 μg per 100 mm dish, 72 h) followed by FLAG immunoprecipitation and western blot demonstrated that NRDP1 can bind to the androgen receptor (AR) in LNCaP cells. The ability of the ΔCC construct, which is missing the NRDP1 coiled coil domain, to mediate AR pulldown indicates that the coiled coil domain is not required for interaction between AR and NRDP1. Detailed information about Western Blot can be found at supplementary materials.

We demonstrate that all three NRDP1 constructs can translocate to the nucleus in LNCaP cells (Figure 4B, full blot; Figure S10). It is noteworthy that for this and other experiments, endogenous NRDP1 is barely detectable and the WT NRDP1 construct is often expressed at lower levels compared to the CHSQ and ΔCC constructs. This is due to the fact that wildtype NRDP1 can very efficiently auto-ubiquitinate and thereby rapidly reduce WT NRDP1 expression levels, while NRDP1-CHSQ and NRDP1-ΔCC cannot efficiently auto-ubiquitinate due to point mutations in the receptor binding domain and removal of the coiled coil domain, respectively, and hence their levels remain high [34]. Immunoprecipitation studies using the NRDP1 constructs determined that NRDP1 can bind to AR in LNCaP cells and that this interaction does not require the coiled coil region of NRDP1 (Figure 5, full blot; Figure S11). All three constructs, including ΔCC which is missing the coiled coil domain, were able to mediate pulldown of AR in LNCaP cells, indicating that the coiled coil domain is not required for interaction of NRDP1 with AR.

**Figure 6:**
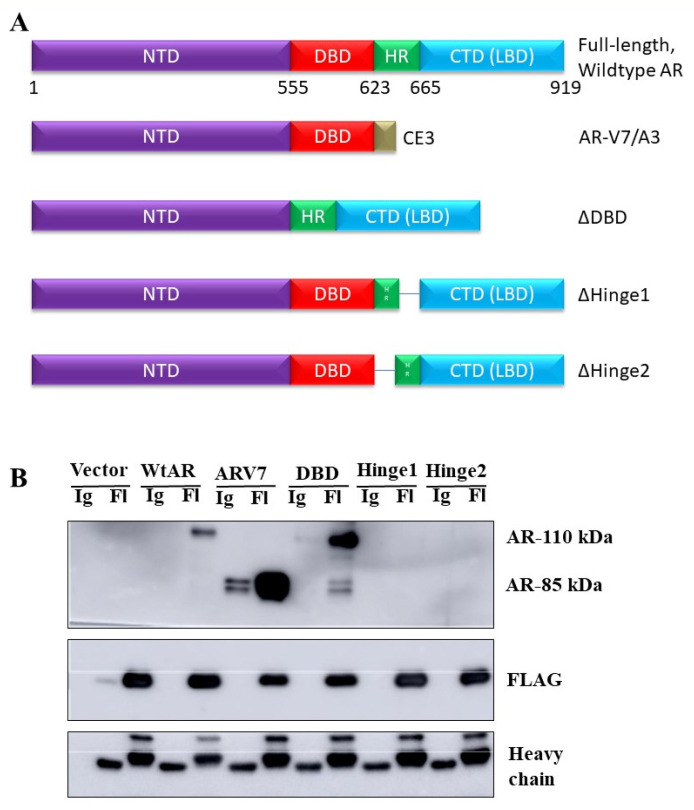
The interaction between NRDP1 and the androgen receptor is dependent on the hinge domain of the androgen receptor. Four androgen receptor (AR) constructs were used to validate the interaction between AR and NRDP1 and to help determine which regions of AR are involved in mediating the interaction; AR V7 (lacks the ligand binding domain (LBD)), ΔDBD (lacks the DNA binding domain (DBD)), ΔHinge 1 and ΔHinge 2 (both constructs lack the hinge region) (**A**). Co-transfection of PC-3 cells (which lack endogenous AR) with the AR constructs and the FLAG-tagged NRDP1-CSHQ construct (3 μg per 100 mm dish, 72 h) followed by FLAG immunoprecipitation and western blot confirmed that interaction between AR and NRDP1 is possible (**B**) pulldown of both the ARV7 and DBD AR constructs was observed. Pulldown of the Hinge constructs was not observed indicating that interaction between AR and NRDP1 may occur via the AR Hinge domain. Detailed information about Western Blot can be found at supplementary materials.

Immunoprecipitation studies were also conducted using the CHSQ construct and various AR constructs to determine which region of AR is required for the NRDP1-AR interaction in LNCaP cells. AR has four major domains (Figure 6A): The N-terminal domain (NTD, required for regulation of AR transcriptional activity and mediates many protein-protein interactions), the DNA binding domain (DBD, directly binds to AR response elements), and the hinge region (modifications to this region allow for integration of signals from various pathways and it thereby regulates transcriptional targets and activity; the hinge region can be acetylated, phosphorylated, and/or methylated) [29]. The following AR constructs were used; AR V7 (lacks the LBD), ΔDBD (lacks the DBD), ΔHinge 1 and ΔHinge 2 (both constructs lack the hinge region). CHSQ FLAG-mediated pull-down of AR was observed when LNCaP cells were co-transfected with CHSQ-FLAG and either the wildtype AR, AR V7, or DBD constructs (Figure 6B, full blot; Figure S12). Note that a blot showing levels of AR in the pre-IP lysate is provided as Figure S13. Pull-down was not observed for the Hinge constructs indicating that the Hinge region is required for interaction between NRDP1 and AR in LNCaP cells. Taken together the above data demonstrated that in LNCaP cells, but not in MDA-PCa-2b, NRDP1 translocation to the nucleus is mediated by binding of NRDP1 to the AR hinge domain.

### 3.6. NRDP1 Can Act as a Ubiquitin Ligase in Both the Cytosol and the Nucleus of CaP Cells

**Figure 7:**
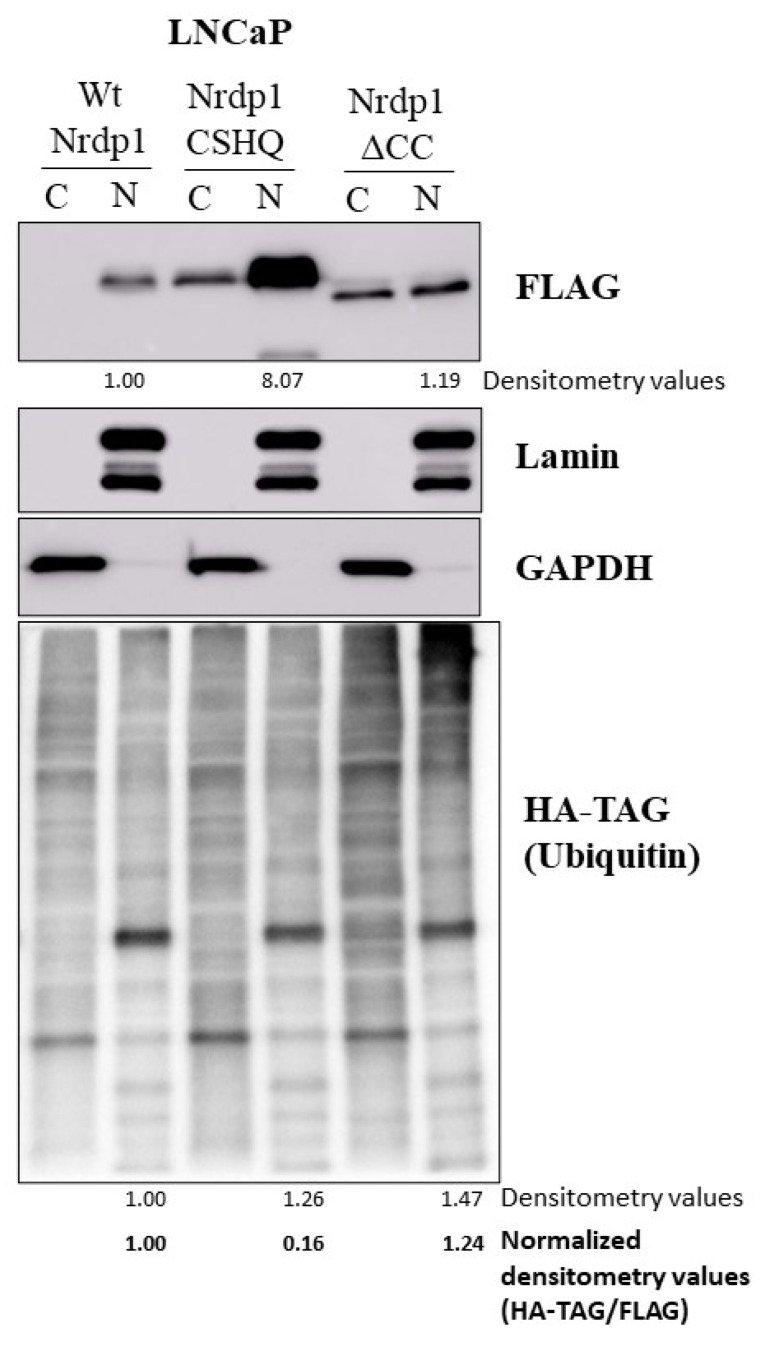
NRDP1 can act as a ubiquitin ligase within the nucleus of prostate cancer cells. Co-transfection of LNCaP with the Wt, CSHQ, or ΔCC NRDP1 constructs with an HA-tagged ubiquitin construct (HA-TAG)(3 ug per 100 mm dish, 72 h) followed by subcellular fractionation and western analyses confirmed that NRDP1 retains its ability to function as a ubiquitin ligase when located in the nucleus; transfection with ΔCC, which retains its ability to ubiquitinate target proteins, caused a 1.24-fold increase in levels of ubiquitinated nuclear proteins compared to Wt NRDP1 (HA-Ub densitometry scores were normalized based on NRDP1-FLAG expression, higher levels of ΔCC NRDP1 were expressed compared to Wt NRDP1 since ΔCC cannot mediate oligomerization/degradation of NRDP1 by auto-ubiquitination). Transfection with CSHQ, caused a 5-fold decrease in levels of ubiquitinated nuclear proteins compared to Wt NRDP1, this is expected as the CSHQ construct cannot ubiquitinate target proteins and would compete with endogenous NRDP1 for target binding. Detailed information about Western Blot can be found at supplementary materials.

To determine if NRDP1 retains its ubiquitin ligase activity in the nucleus, LNCaP cells were co-transfected with wildtype NRDP1, NRDP1-CSHQ, or the NRDP1-ΔCC constructs and HA-tagged ubiquitin (HA-Ub) and separated into nuclear and cytoplasmic fractions (Figure 7, full blot; Figure S14). As would be expected, transfection with the NRDP1-CHSQ construct, which is unable to efficiently ubiquitinate binding partners, did not result in a significant increase in the level of ubiquitinated proteins in the cytosolic or nuclear fractions compared to wildtype NRDP1; normalization of nuclear ubiquitinated protein levels based on NRDP1 construct level shows that transfection with NRDP1-CSHQ resulted in a 6.25-fold decrease in nuclear ubiquitinated protein levels compared to transfection with NRDP1-WT. In contrast, transfection with the NRDP1-ΔCC construct, which can ubiquitinate target proteins but cannot auto-ubiquitinate, resulted in a 1.24-fold increase in nuclear ubiquitinated protein levels compared to transfection with NRDP1-WT. Note that the ability of Nrdp-WT and NRDP1-ΔCC to bind to and ubiquitinate proteins was confirmed by co-transfection of NRDP1 constructs and HA-ubiquitin followed by immunoprecipitation (Figure S3); Nrdp-WT and NRDP1-ΔCC both pulled down ubiquitinated proteins while NRDP1-CHSQ did not. The combined data indicate that NRDP1 can act as a ubiquitin ligase in the nucleus as well as the cytosol of CaP cells.

### 3.7. Nuclear NRDP1 Levels Are Regulated by Proteasomal Degradation in Some but Not All Prostate Cancer Cells

**Figure 8:**
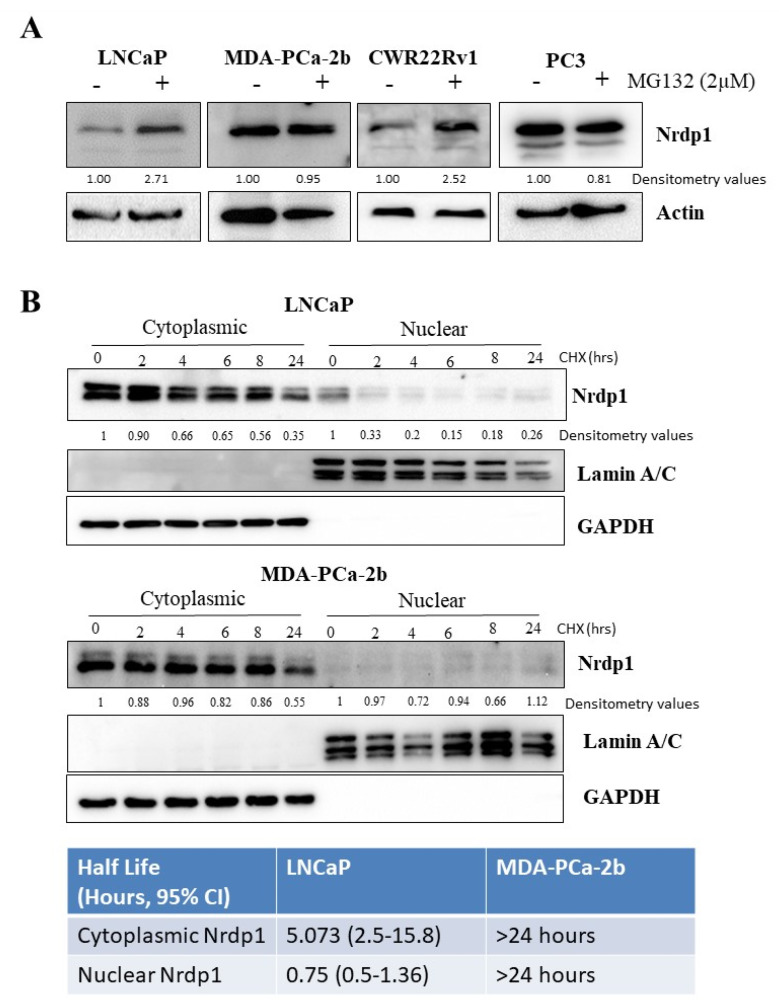
Nuclear NRDP1 levels are regulated by proteasomal degradation in some but not all prostate cancer cells. Treatment of LNCaP, MDA-Pca-2b, and CWR22Rv1 cells with a proteasome inhibitor, MG-132 (2 μM, 16 h), resulted in accumulation of NRDP1 indicating that proteasomal degradation can regulate NRDP1 levels in these cell lines (**A**); 1.85, 1.76, and 2.52-fold increases in NRDP1 levels following MG-132 treatment (normalized to actin). Treatment with cycloheximide, which inhibits protein synthesis (100 μg/mL), over a 24-h period followed by subcellular fractionation and western analyses revealed that nuclear NRDP1 has a much shorter half-life compared to cytoplasmic NRDP1 in LNCaP cells (**B**); the nuclear half-life of NRDP1 was approximately 0.75 h compared to a cytoplasmic half-life of approximately 5 h. In MDA-Pca-2b, there was little or no decrease in nuclear or cytoplasmic NRDP1 levels over the 24-h time period indicating that stabilization is occurring. Detailed information about Western Blot can be found at supplementary materials.

Wildtype NRDP1 is known to auto-ubiquitinate and can then undergo proteasomal degradation [32]. Inhibition of proteasome function using MG-132 resulted in accumulation of endogenous NRDP1 in LNCaP and CWR22Rv1 cells (1.85, 1.76, and 2.12-fold increase (normalized to actin), respectively) but not in PC3 cells (Figure 8A, full blot; Figure S15), indicating that proteasomal degradation may be a mechanism by which NRDP1 protein levels may be regulated in some but not all CaP cells. To further understand the role of protein degradation in regulating NRDP1 levels we treated LNCaP and MDA-PCa-2b cells with the protein synthesis inhibitor cycloheximide which prevents *de novo* protein synthesis. Half-life of endogenous cytosolic and nuclear NRDP1 in these cells was calculated based on the changing levels of the protein over time following cycloheximide treatment (Figure 8B, full blots; Figures S16 and S17). In LNCaP cells, the half-life of cytosolic NRDP1 was approximately 5 h, whereas the half-life of nuclear NRDP1 was less than 1 h. In MDA-PCa-2b cells, we determined that cytosolic NRDP1 was very stable (half-life > 24 h). It is noteworthy that we also observed stabilization of Nrdp1 in CWR22Rv1 cells, a castration resistant cell line (Figure S4). In alignment with our previous experiments, very low levels of endogenous nuclear NRDP1 were observed and there appeared to be little or no decrease in nuclear NRDP1 levels over the 24-h time course. These data indicate that increased rate of degradation via the proteasome is unlikely to be responsible for the lower level of nuclear NRDP1 that is observed in MDA-PCa-2b cells compared to LNCaP cells.

## 4. Discussion

Our data confirm that NRDP1 can be found in both the nucleus and cytoplasm of prostate cancer cells. We show that NRDP1 retains its ability to function as a ubiquitin ligase within the nucleus and that AR appears to play a key role in mediating nuclear translocation of NRDP1. The association between nuclear NRDP1 levels and PSA recurrence in prostate cancer patients indicates that nuclear NRDP1 has clinical relevance and that further investigation of how NRDP1 translocation occurs and how nuclear NRDP1 levels are regulated as well as the identification of specific nuclear targets of nuclear NRDP1 is warranted.

While nuclear localization of NRDP1 in patient CaP cells has not been reported by other groups, this finding aligns with the TCGA data which shows increased NRDP1 mRNA levels are associated with decreased survival rates for CaP patients (Figure S5) [35]. Our in vitro data also support a link between increased nuclear NRDP1 expression and CaP disease progression; CRPC cell lines expressed much higher levels of nuclear NRDP1 compared to androgen sensitive cell lines. Nuclear NRDP1 has been reported in invasive breast cancer biopsy cells indicating that nuclear NRDP1 may play a role in other cancer types [6]. An association between NRDP1 levels and recurrence-free progression was not observed in the breast cancer study, however, total NRDP1 levels not nuclear NRDP1 levels were assessed meaning it is still possible that an association between nuclear NRDP1 and patient outcomes exists in this setting.

To our knowledge, no other study has focused on determining how nuclear translocation of NRDP1 occurs, how nuclear NRDP1 levels are regulated, or whether nuclear NRDP1 retains its ubiquitin ligase activity. NRDP1 does not have a nuclear localization signal (NLS) and therefore must be dependent on another molecule for entry into the nucleus. Our patient and in vitro data indicate that the AR plays a key role in mediating NRDP1 nuclear translocation; a statistically significant correlation between AR and NRDP1 localization was found in patient biopsy samples, and our in vitro studies demonstrate that AR can bind to NRDP1 and that manipulation of AR expression and activity can impact NRDP1 cellular localization. In further support of AR playing a role, AR has been shown to mediate the nuclear transport of other molecules which lack a nuclear transport signal, for example beta-catenin [21]. Beta-catenin binds to the ligand binding domain (LBD) of AR and once co-translocated into the nucleus it can serve as a co-factor and regulate AR transcriptional activity [36,37,38]. We demonstrate that binding of AR to NRDP1 does not require the coiled coil domain of NRDP1, the domain which allows for NRDP1 oligomerization and auto-ubiquitination, and does require the hinge domain of AR. Modifications to the hinge region of AR (e.g., acytylation, phosphorylation, methylation) are known to regulate binding of AR to transcriptional targets [39], and along with the AR DBD it also houses the bipartite NLS which is required for AR translocation [39,40]. Goals for future studies include determination of the exact sites required for binding between NRDP1 and AR as well as to determine whether inhibition of binding impacts AR transcriptional activity and/or the ability of NRDP1 and/or AR to translocate to the nucleus. Inhibiting AR translocation and/or transcriptional activity are key therapeutic strategies for the treatment of recurrent CaP [41,42,43].

Our finding that NRDP1 can act as an E3 ubiquitin ligase within the nucleus is somewhat expected based on the fact that many other types of nuclear ubiquitin ligases play a key role in the regulation of nuclear protein levels [44]. For example, MDM2 can cause ubiquitination and degradation of nuclear p53 [45]. The specific targets of nuclear NRDP1 remain to be identified and this will be a key focus of future studies. Potential targets which are known to be regulated by ubiquitination and play a key role in driving CaP progression include PTEN and AR [27,28,46]. Nrdp1-mediated degradation of AR would help explain why a correlation between nuclear AR and Nrdp1 was not observed in our patient study. As with many other ubiquitin ligases, NRDP1 is able to autoubiquitinate and this is a key mechanism by which NRDP1 levels are regulated [34]. USP8, a deubiquitinase, counters NRDP1 autoubiquitination and thereby increases NRDP1 levels [32]. Inhibition of USP8 has been shown to decrease NRDP1 levels and decrease cell proliferation and drug resistance in several cancers including breast, lung, and cervical cancer [47]. For example, knockdown of USP8 in lung cancer cells inhibited the proliferation of lung cancer cells by regulating several cell cycle and apoptosis-related proteins [48]. BRUCE (also known as BIRC6), an IAP, is a key regulator of USP8 activity. While the relationship between BRUCE, USP8, and NRDP1 has not been established in cancer cells, it is noteworthy that elevated levels of BRUCE are associated with worse outcomes in CaP patients [49]. If the relationship between BRUCE, USP8, and NRDP1 which has been reported in other cell types exists in CaP cells then elevated levels of BRUCE would potentially result in increased levels of NRDP1 and thereby align with our patient data.

In our previous studies, we found an association between overall levels of Nrdp1 and levels of ErbB3; decreased expression of total levels of Nrdp1 resulted in increased levels of ErbB3 and increased CaP cell proliferation [7]. We now show that an increase in nuclear Nrdp1 is associated with worse patient outcome. Based on our finding that nuclear Nrdp1 retains its ubiquitin ligase activity, it is possible that nuclear Nrdp1 targets nuclear tumor suppressors for degradation thereby driving disease progression. Our on-going studies are focused on identifying and validating nuclear targets to further investigate this possibility.

Our cell line data indicate that NRDP1 levels are regulated by proteasomal degradation in some but not all CaP cell lines. We also found a difference between the half-life of cytoplasmic and nuclear NRDP1 with the latter being much longer. In addition to identifying the targets of nuclear NRDP1 and determining the precise mechanism by which NRDP1 nuclear translocation occurs in CaP cells, we also plan to further elucidate the mechanisms by which nuclear NRDP1 levels are regulated. While the degree of nuclear translocation will contribute to this, we hypothesize that molecules such as USP8 and BRUCE also play a role. It is noteworthy that BRUCE plays a key role in autophagy regulation and as such can direct degradation of NRDP1 via lysosomes [50,51]. This may help explain why we do not see proteasomal degradation of NRDP1 occurring in some CaP cell lines.

## 5. Conclusions

The combined data support a role for nuclear NRDP1 in mediating CaP progression. Further understanding of the mechanism by which nuclear translocation occurs and how nuclear NRDP1 levels are regulated as well as identification of targets of nuclear NRDP1 could improve our understanding of CaP pathogenesis and support the development of drugs for the treatment of CaP and other cancers and disease states for which dysregulation of NRDP1 plays a role.

## Figures and Tables

**Table 1:** Patient characteristics.

Characteristics	Number
Age	
*N*	162
Mean (SD)	69.7 (8.2)
Median (Range)	69 (49–91)
Weight (lbs)	
*N*	160
Mean (SD)	181.6 (47.5)
Median (Range)	185.9 (64.9–284)
Race	
White	102 (63%)
Black/African-American	31 (19.1%)
American Indian/AK Native	3 (1.9%)
Asian	1 (0.6%)
Unknown	25 (15.4%)
Ethnicity	
Not Hispanic or Latino	138 (85.2%)
Hispanic or Latino	4 (2.5%)
Unknown	20 (12.3%)
Smoking	
Current/Former Smoker	95 (58.6%)
Never Smoker	67 (41.4%)

**Table 2:** Logistic regression models of PSA failure by Marker Expression.

Marker	Odds Ratio (95% CI)	*p*-Value
AR.Cancer.N	0.935 (0.845, 1.034)	0.193
AR.Cancer.C	1.153 (0.708, 1.876)	0.568
Ki67.Cancer	0.998 (0.997, 1)	0.0477
NRDP1.Cancer.N	1.238 (1.063, 1.443)	0.00684
NRDP1.Cancer.C	1 (0.81, 1.235)	1

Odds ratio = change in odds of PSA failure for unit increase in marker. N, nuclear localization; C, cytoplasmic localization.

**Table 3:** Recurrence Free Percentages at Five Years by Marker Expression.

Marker	5-Year Recurrence-Free Percentage, High Expression	5-Year Recurrence-Free Percentage, Low Expression	Difference (95% CI)	*p*-Value
AR N	74.50%	87.10%	−12.7% (−31.8%, 6.5%)	0.196
AR C	95.50%	79.10%	16.4% (7.5%, 25.2%)	<0.001
Ki67	77.40%	94.40%	−17% (−27.1%, −6.9%)	0.001
NRDP1 N	73.50%	92%	−18.5% (−30.9%, −6.1%)	0.003
NRDP1 C	85.10%	87.40%	−2.3% (−12.3%, 7.7%)	0.650

N, nuclear localization; C, cytoplasmic localization; Bolded font denotes statistically significant differences.

**Table 4:** Correlations between AR, NRDP1, and Ki67 expression in prostate cancer tumor cells from patients with stage 1/2 disease. N, nuclear localization; C, cytoplasmic localization. Bolded font denotes statistically significant correlations.

Marker 1	Marker 2	Spearman Correlation	*p*-Value
AR N	AR C	0.32	<0.001
AR N	Ki67	0.22	0.012
AR N	NRDP1 N	−0.11	0.216
AR C	Ki67	0.06	0.525
AR C	NRDP1 N	−0.46	<0.001
Ki67	NRDP1 N	−0.06	0.476

**Table 5:** Correlations between AR, NRDP1, and Ki67 expression in prostate cancer tumor cells from patients with stage 3 disease. N, nuclear localization; C, cytoplasmic localization. Bolded font denotes statistically significant correlations.

Marker 1	Marker 2	Spearman Correlation	*p*-Value
AR N	AR C	0.03	0.790
AR N	Ki67	0.51	<0.001
AR N	NRDP1 N	0.07	0.564
AR C	Ki67	0	0.974
AR C	NRDP1 N	−0.35	0.003
Ki67	NRDP1 N	0.18	0.128

## Data Availability

TCGA database; https://www.cancer.gov/about-nci/organization/ccg/research/structural-genomics/tcga, accessed on 20 July 2021 [35].

## Supplementary Materials

The following are available online at https://www.mdpi.com/article/10.3390/cancers13174425/s1. Figure S1: Table showing the relationship between Nrdp1 and Gleason score; Figure S2: Nrdp1 and PSA reporter gene assay showing that Nrdp1 expression is associated with AR activity; Figure S3: Co-transfection with various NRDP1-FLAG construct and HA-Ubiquitin followed by pulldown with FLAG; Figure S4: Cyclohexamide experiment data comparing Nrdp1 half-life in CWR22Rv1, LNCaP, and MDA-PCa-2b cells; Figure S5:TCGA data show that higher levels of NRDP1 expression are associated with worse outcomes for prostate cancer patients; Figure S6: Full blots corresponding to Figure 1B; Figure S7: Full blots corresponding to Figure 3A; Figure S8: Full blots corresponding to Figure 3B; Figure S9: Full blots corresponding to Figure 3C; Figure S10: Full blots corresponding to Figure 4B; Figure S11: Full blots corresponding to Figure 5; Figure S12: Full blots corresponding to Figure 6B; Figure S13: Western blot showing levels of AR in the lysate prior to immunoprecipitation for Figure 6B; Figure S14: Full blots corresponding to Figure 7 (with HA-TAG full blot which is already shown in Figure 7); Figure S15: Full blots corresponding to Figure 8A; Figure S16: Full blots corresponding to Figure 8B, upper panel. Figure S17: Full blots corresponding to Figure 8B, lower panel.
