# Supplementary material for: Androgen Receptor-Mediated Nuclear Transport of NRDP1 in Prostate Cancer Cells Is Associated with Worse Patient Outcomes
Source: Cancers (Basel). 2021 Sep 2;13(17):4425. doi: 10.3390/cancers13174425 (PMC8430998; doi:10.3390/cancers13174425)
Supplement: Supplementary file 1 [file cancers-13-04425-s001.zip › cancers-1342986-supplementary.pdf]

Supplementary Materials

Androgen Receptor-Mediated Nuclear Transport of NRDP1 in Prostate Cancer Cells Is Associated with Worse Patient Outcomes

Thomas Steele, Anhao Sam, Shawna Evans, Elizabeth Browning, Sheryl Krig, Katelyn Macias, Adarsh Konda, Salma Siddiqui, Blythe Durbin-Johnson, Paramita Ghosh and Ruth Vinall

Marker	Spearman Correlation	P-Value
AR N	0.28	<0.001
AR C	0.14	0.012
Ki67	0.37	<0.001
NRDP1 N	0.02	0.836
NRDP1 C	0.11	0.179

Figure S1. Correlation between NRDP1 and Gleason score. N = nuclear localization, C = cytoplasmic localization. Bolded font denotes statistically significant correlations.

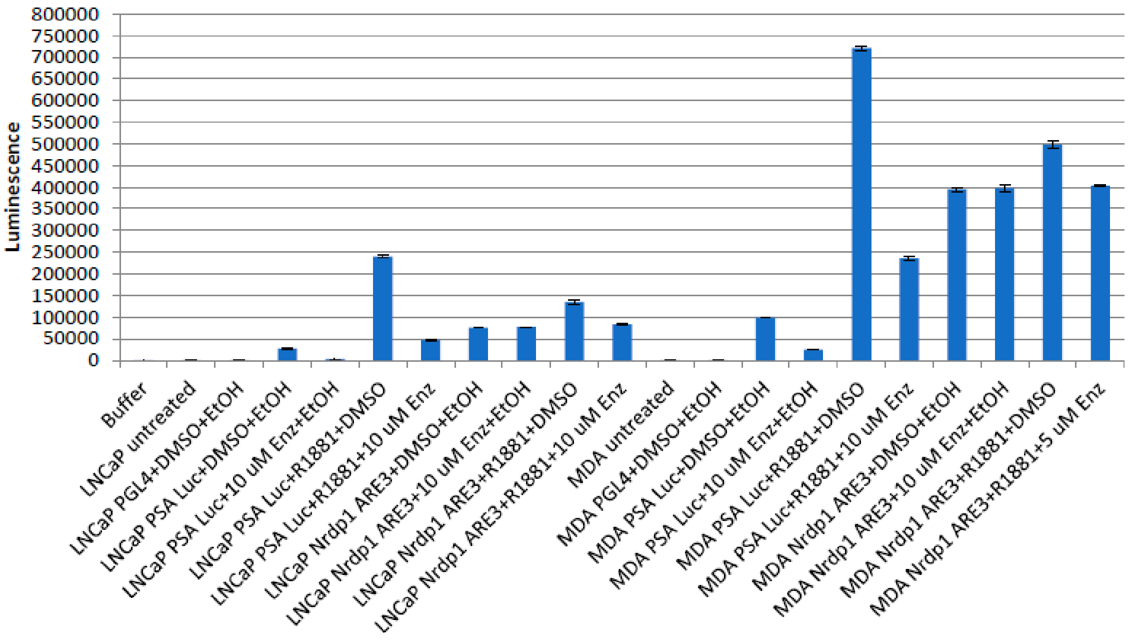

Figure S2. Androgen-induced transcriptional activity of the PSA and Nrdp1 genes in LNCaP and MDA-PCa-2b can be inhibited by treatment with enzalutamide. Reporter gene assay data (48 hours posttreatment) show that the MDA-PCa-2b cell line has higher basal activity of the PSA gene (MDA-PCa-2b activity is ~4-fold higher compared to LNCaP) and the Nrdp1 gene (MDA-PCa-2b activity is ~5-fold higher compared to LNCaP). Treatment with synthetic androgen (R1881, 1 nM) caused an increase in transcriptional activity of the PSA gene in both cell lines (~9-fold increase in LNCaP, and ~7-fold increase in MDA-PCa-2b), and an increase in Nrdp1 gene transcriptional activity in MDA-PCa-2b (~1.8-fold increase in LNCaP, and a ~1.25-fold increase in MDA-PCa-2b). Treatment with enzalutamide (enz, 10 uM) caused a reduction in both PSA and Nrdp1 expression in LNCaP (~2-fold decrease) and in MDA-PCa-2b (1.25-fold decrease), further indicating that the androgen receptor can regulate Nrdp1 expression in both cell lines.

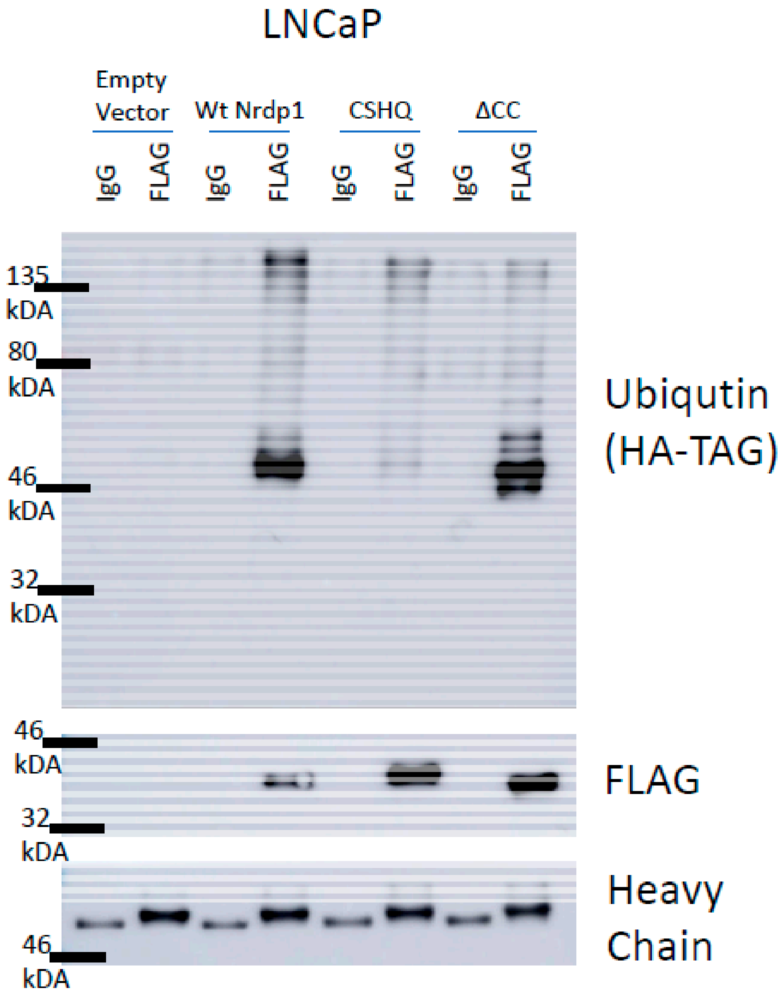

Figure S3. Co-transfection with various Nrdp1-FLAG construct and HA-Ubiquitin followed by pulldown with FLAG.

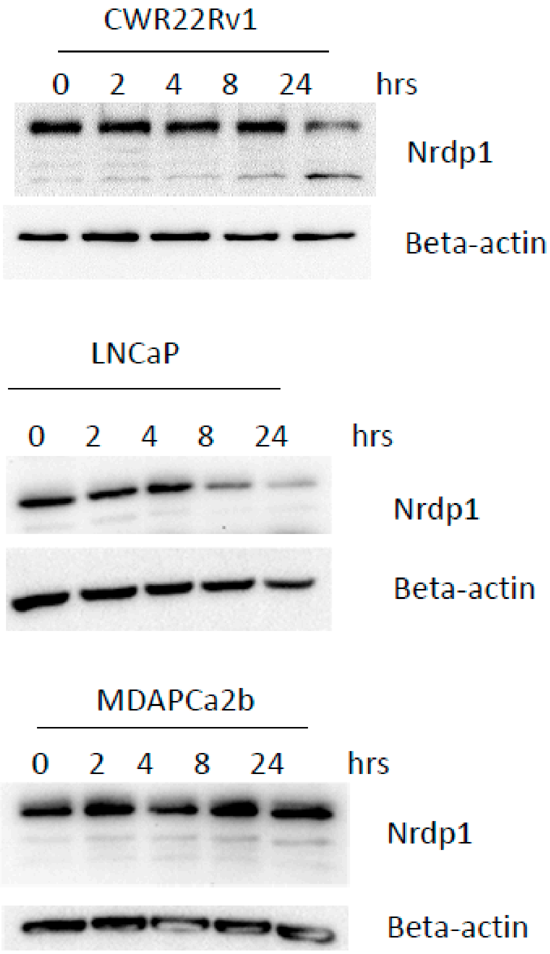

Figure S4. Different prostate cancer cell lines exhibit different Nrdp1 protein half-lives. CWR22Rv1, a castration resistant cell line, and MDA-PCa-2b, an androgen sensitive cell line, exhibited longer Nrdp1 protein half-lives compared to LNCaP, an androgen sensitive cell line. The variation between half-lives indicates that different prostate cancer cells may have different dependencies on Nrdp1.

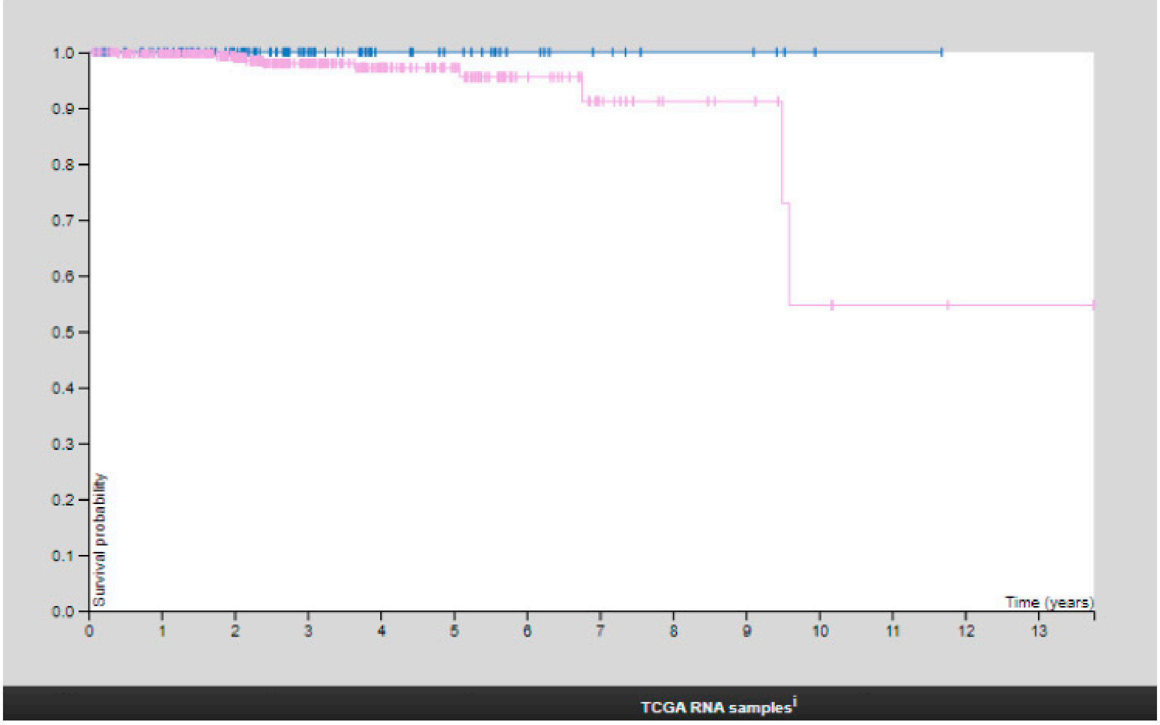

Blue line = Low expression of Nrdp1 (n=119)
Purple line = High expression of Nrdp1 (n=375)

Figure S5. TCGA data show that higher levels of Nrdp1 expression are associated with worse outcomes for prostate c ancer patients. ICGC/TCGA Pan-Cancer Analysis of Whole Genomes Consortium. Pan-cancer analysis of whole genomes. Nature. **2020**, *578*, 82–93. doi: 10.1038/s41586-020-1969-6. Epub 2020 Feb 5. PMID: 32025007; PMCID: PMC7025898.

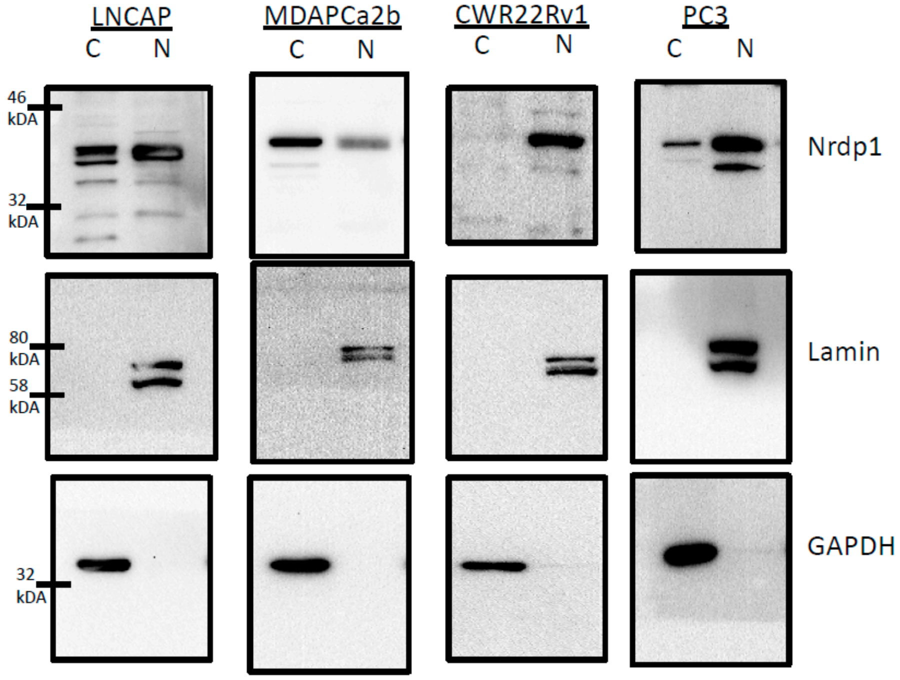

Figure S6. Full blots corresponding to Figure 1B.

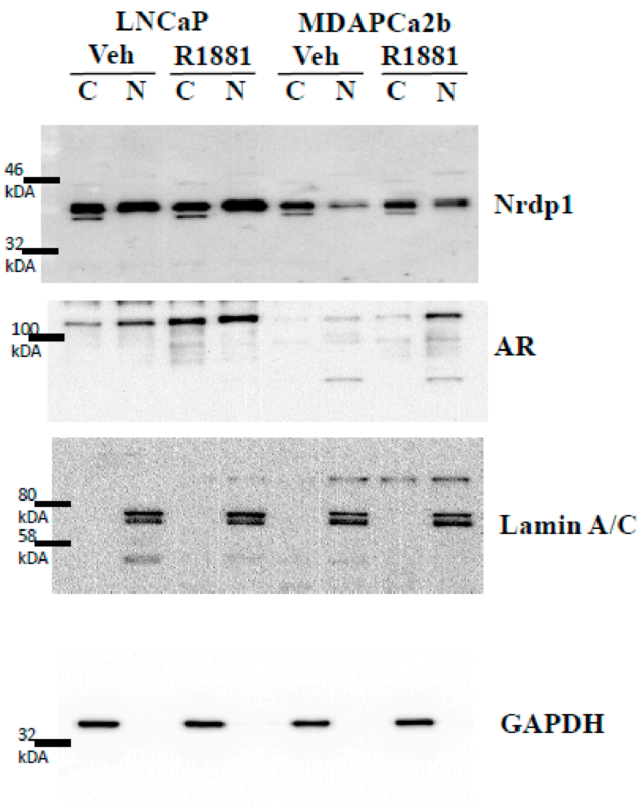

Figure S7. Full blots corresponding to Figure 3A.

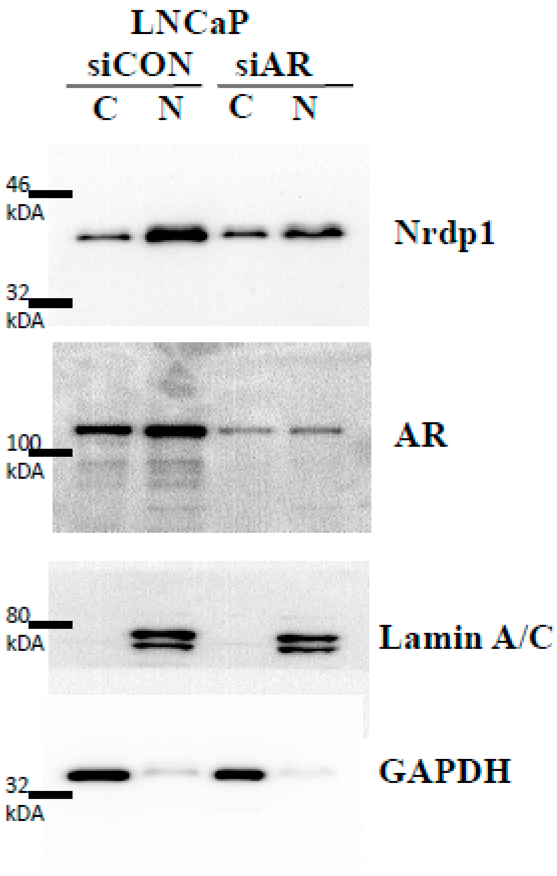

Figure S8. Full blots corresponding to Figure 3B.

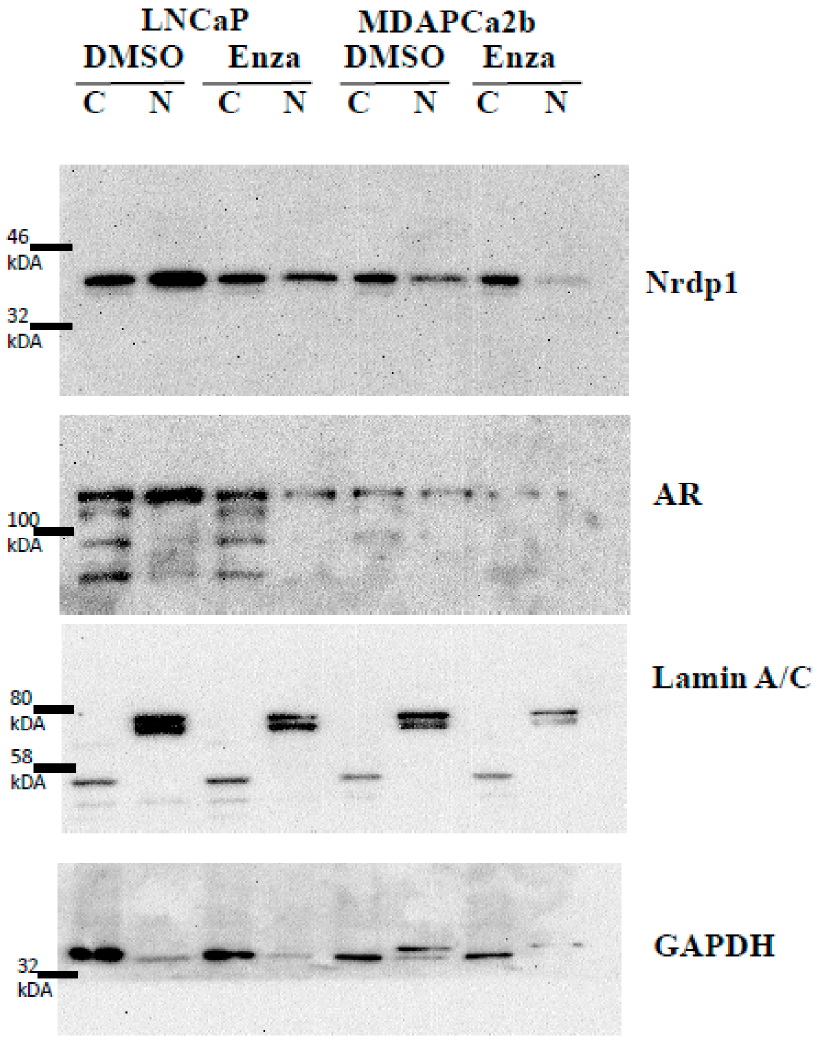

Figure S9. Full blots corresponding to Figure 3C.

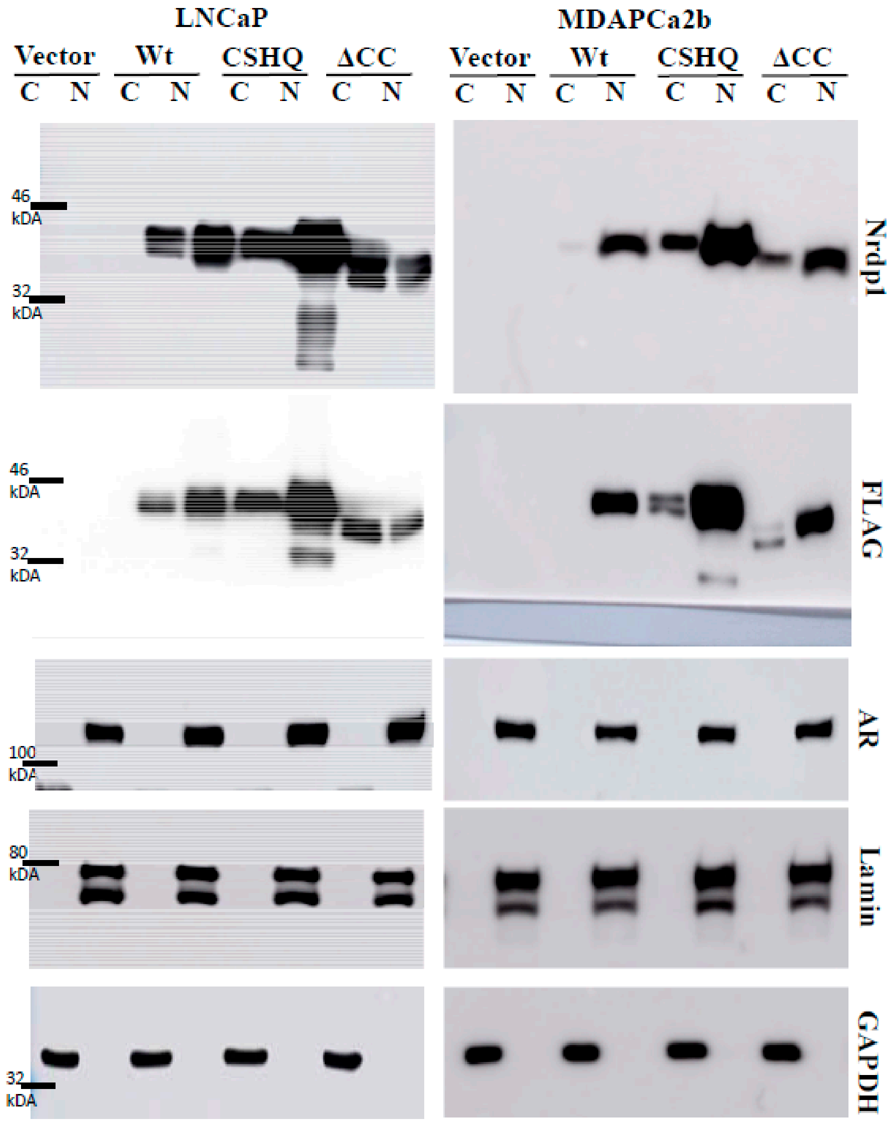

Figure S10. Full blots corresponding to Figure 4B.

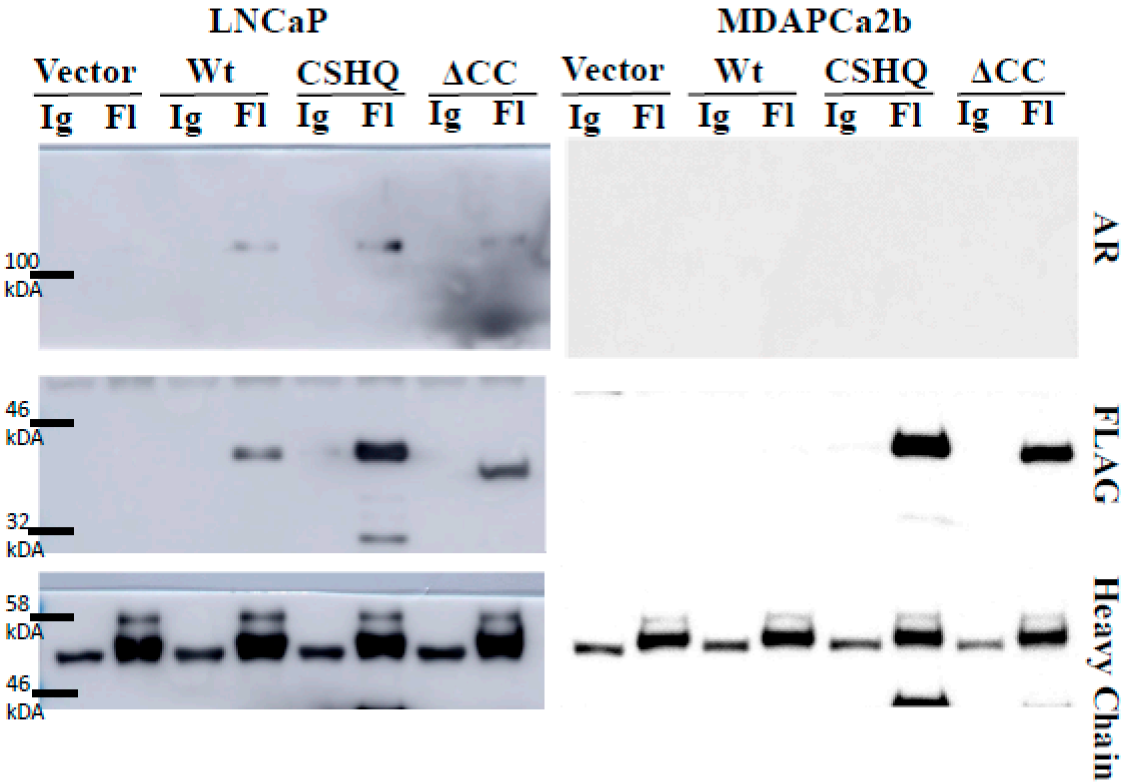

Figure S11. Full blots corresponding to Figure 5.

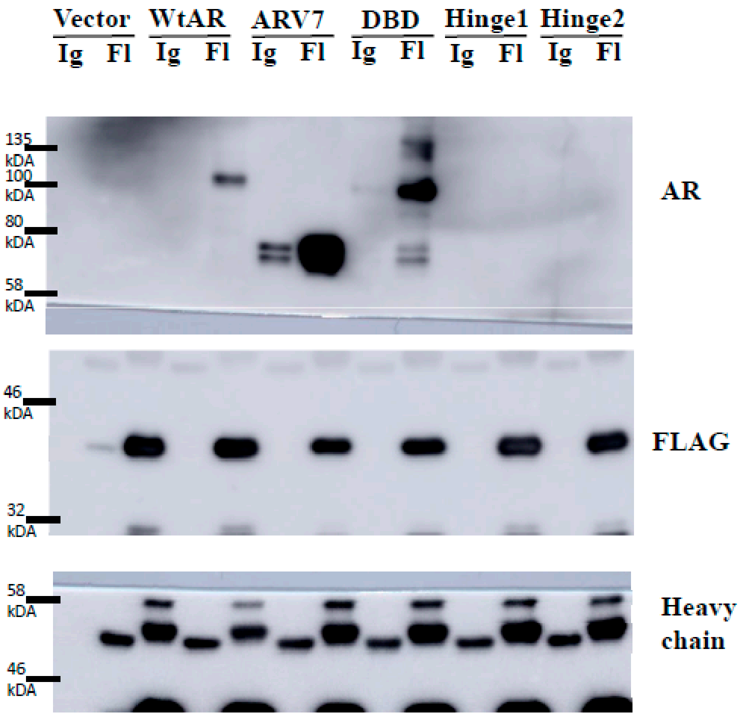

Figure S12. Full blots corresponding to Figure 6B.

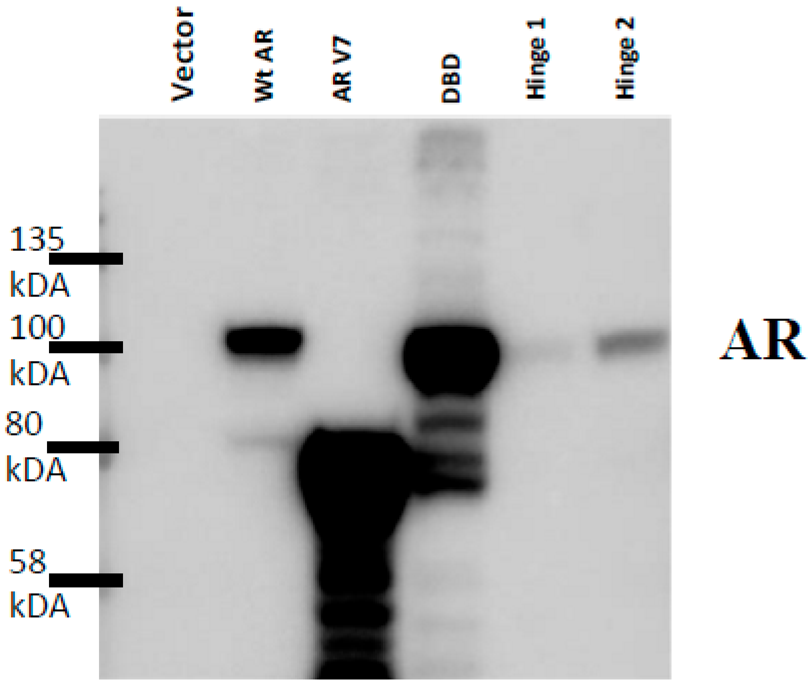

Figure S13. Western blot showing levels of AR in the lysate prior to immunoprecipitation for Figure 6B.

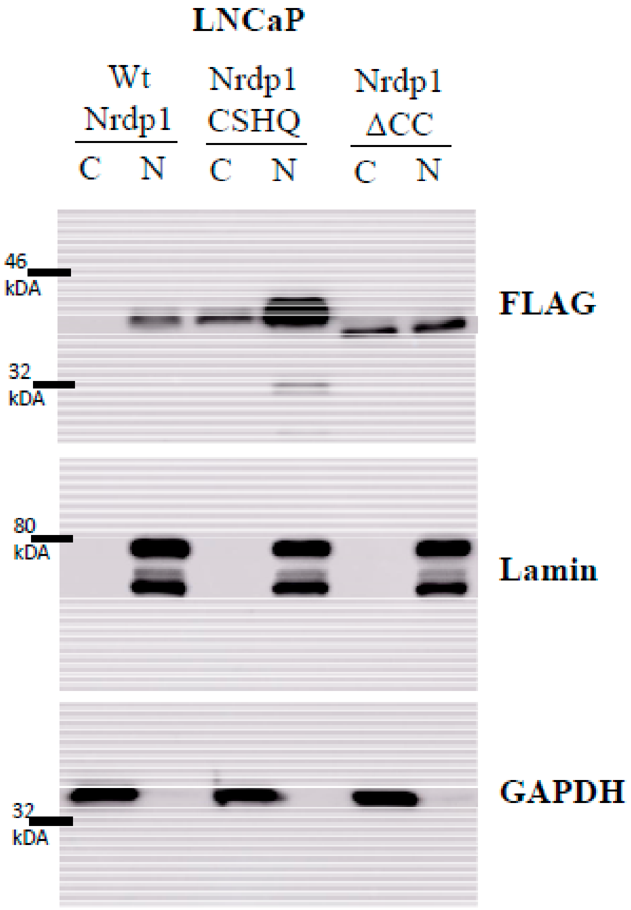

Figure S14. Full blots corresponding to Figure 7 (with HA-TAG full blot which is already shown in Figure 7).

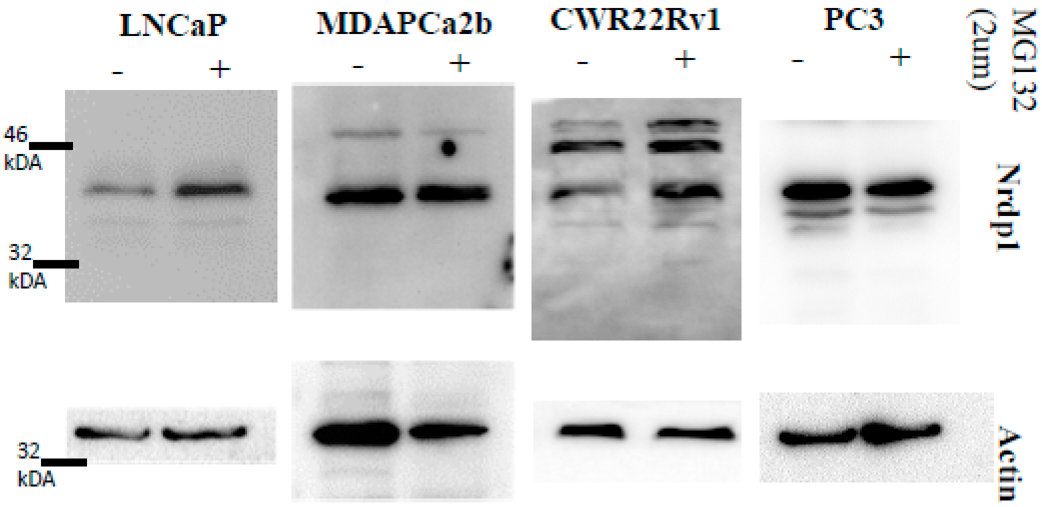

Figure S15. Full blots corresponding to Figure 8A.

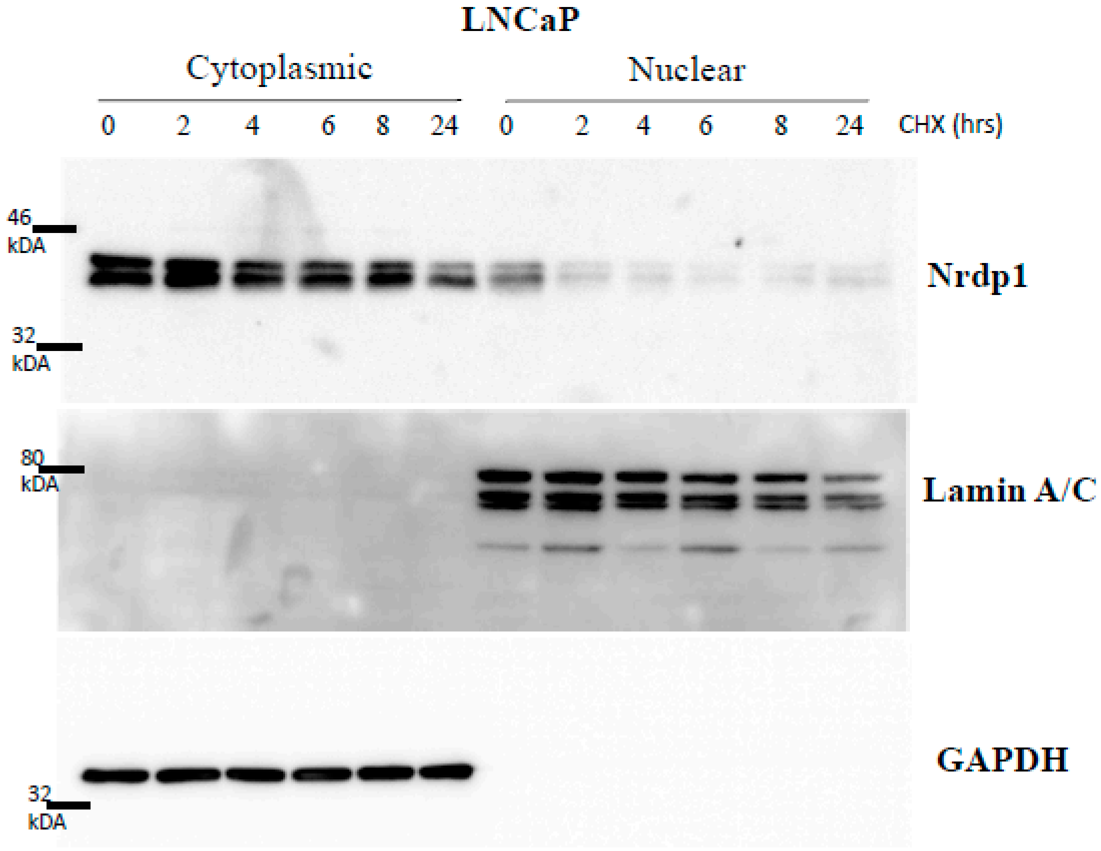

Figure S16. Full blots corresponding to Figure 8, upper panel.

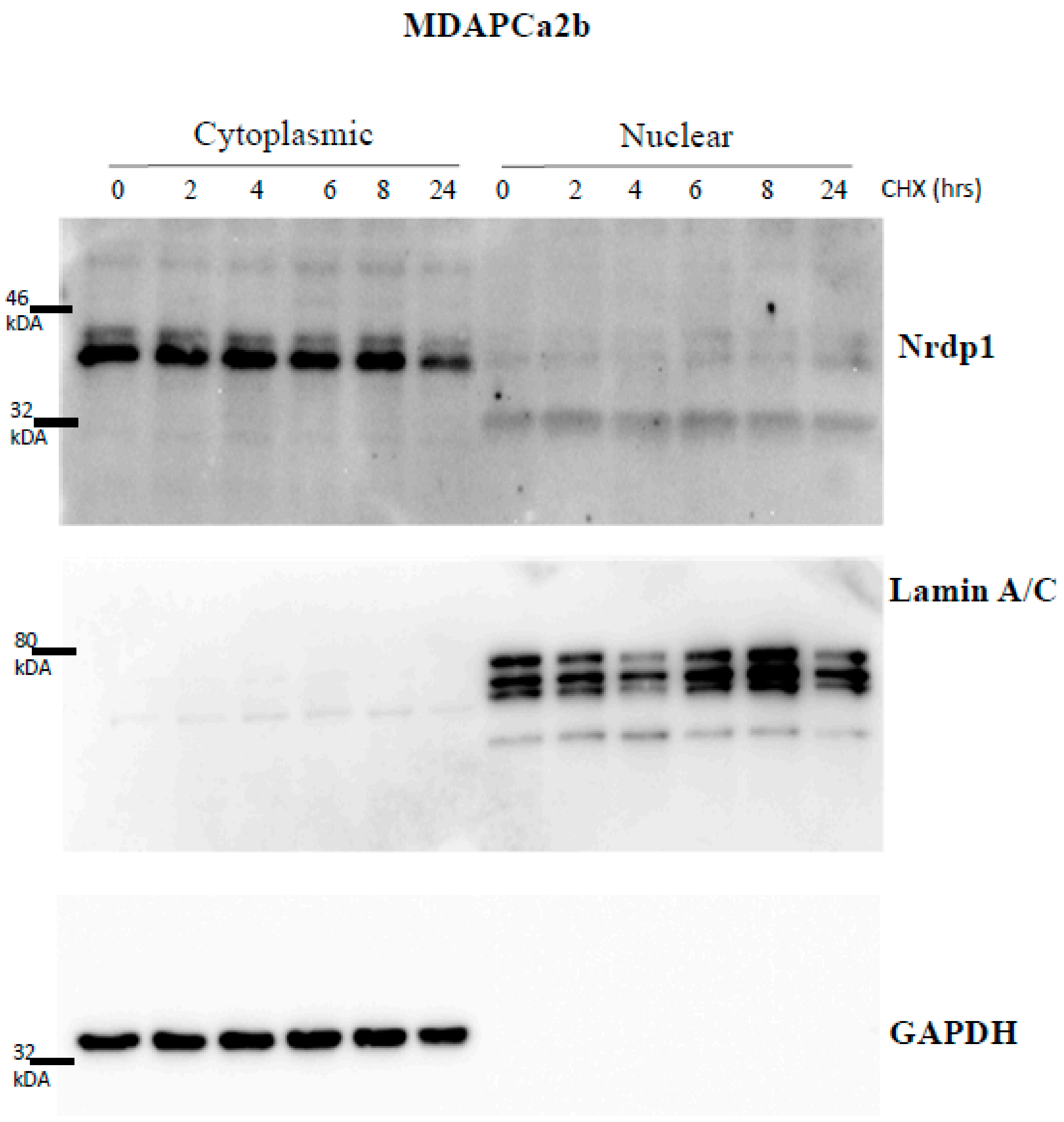

Figure S17. Full blots corresponding to Figure 8B, lower panel.
